# Striatal GABA levels correlate with risk sensitivity in monetary loss

**DOI:** 10.3389/fnins.2024.1439656

**Published:** 2024-07-31

**Authors:** Hirohito M. Kondo, Takeyuki Oba, Takahiro Ezaki, Takanori Kochiyama, Yasuhiro Shimada, Hideki Ohira

**Affiliations:** ^1^School of Psychology, Chukyo University, Nagoya, Aichi, Japan; ^2^Graduate School of Informatics, Nagoya University, Nagoya, Aichi, Japan; ^3^Precursory Research for Embryonic Science and Technology, Japan Science and Technology Agency, Kawaguchi, Saitama, Japan; ^4^Research Center for Advanced Science and Technology, University of Tokyo, Tokyo, Japan; ^5^Brain Activity Imaging Center, ATR-Promotions, Seika-cho, Kyoto, Japan; ^6^Advanced ICT Research Institute, National Institute of Information and Communications Technology, Osaka, Japan

**Keywords:** decision making, risk, striatum, insula, GABA, learning, monetary task

## Abstract

**Background:**

Decision-making under risk is a common challenge. It is known that risk-taking behavior varies between contexts of reward and punishment, yet the mechanisms underlying this asymmetry in risk sensitivity remain unclear.

**Methods:**

This study used a monetary task to investigate neurochemical mechanisms and brain dynamics underpinning risk sensitivity. Twenty-eight participants engaged in a task requiring selection of visual stimuli to maximize monetary gains and minimize monetary losses. We modeled participant trial-and-error processes using reinforcement learning.

**Results:**

Participants with higher subjective utility parameters showed risk preference in the gain domain (*r* = −0.59) and risk avoidance in the loss domain (*r* = −0.77). Magnetic resonance spectroscopy (MRS) revealed that risk avoidance in the loss domain was associated with γ-aminobutyric acid (GABA) levels in the ventral striatum (*r* = −0.42), but not in the insula (*r* = −0.15). Using functional magnetic resonance imaging (fMRI), we tested whether risk-sensitive brain dynamics contribute to participant risky choices. Energy landscape analyses demonstrated that higher switching rates between brain states, including the striatum and insula, were correlated with risk avoidance in the loss domain (*r* = −0.59), a relationship not observed in the gain domain (*r* = −0.02).

**Conclusions:**

These findings from MRS and fMRI suggest that distinct mechanisms are involved in gain/loss decision making, mediated by subcortical neurometabolite levels and brain dynamic transitions.

## 1 Introduction

Performance of decision making amidst uncertainty is crucial to survive in unknown environments for humans and other animals. We frequently learn values of choices through trial and error in everyday activities, such as business scenes, investments, and purchasing behaviors. In general, risk and uncertainty are distinguished in economics (Knight, 1921). Risk is a situation where the probability that an outcome will occur is known, whereas uncertainty is a situation where its probability is unknown. Although properly assessing and acting on risk is crucial to maximize expected profits, human decision-making often involves interesting behaviors. For example, it is well known that individuals assess their gain and loss perspectives in an asymmetric manner (Kahneman and Tversky, 1979). Given two options, each with certain and probabilistic outcomes that have the same expected value, people tend to avoid risks when gain is expected, but are likely to take risks when loss is expected. This behavior reflects biased information processes under risk situations. However, neural mechanisms for this asymmetric risk sensitivity are poorly understood.

Prospect theory assumes that people give subjective weights to values, i.e., subjective utility, and weights for losses are larger than those for gains, i.e., loss aversion (Kahneman and Tversky, 1979). This theory was refined and developed to provide formal mathematical models for a value function and a probability weighting function (Tversky and Kahneman, 1992). Based on prospect theory, previous studies have investigated neural responses in decision-making under risk (De Martino et al., 2006; Tom et al., 2007). Nilsson et al. (2011) used a hierarchical Bayesian method to estimate model parameters in prospect theory and showed that loss aversion can be predicted by a lower subjective utility for a gain domain than for a loss domain. Prospect theory explains decision-making under risk, but may be silent when values must be learned from experience. Learning of values from experience is often described by reinforcement learning models (Sutton and Barto, 2018). Such models with subjective utility can explain individual differences of risk preference (Niv et al., 2012; Oba et al., 2021). In order to enhance well-being, we need to examine biological factors of individual-level risk-taking propensity. The present study integrates prospect theory, namely, subjective utility, with a reinforcement learning model to identify the process by which risk attitudes are formed through experience.

Many researchers have focused on experience-based choices to investigate brain functions of risk sensitivity in terms of reward valence, e.g., expectation, outcome, and evaluation (Liu et al., 2011). Information on probabilistic outcomes is not always available. Learning progresses when outcomes differ from expectations (Fouragnan et al., 2018). Functional magnetic resonance imaging (fMRI) studies have demonstrated that reward valence is encoded by the ventral striatum (STR; Knutson et al., 2001; Ballard and Knutson, 2009; Talmi et al., 2009; Niv et al., 2012) and the orbitofrontal cortex (OFC; Breiter et al., 2001; Knutson et al., 2003). In addition, previous studies have shown different effects of reward and loss on activation of brain regions, such as the STR, OFC, anterior cingulate cortex (ACC), and insula (INS; Small et al., 2005; Taylor et al., 2006; Wächter et al., 2009; Palminteri et al., 2012; Kim et al., 2015). A recent meta-analysis study indicates that the positive valence network encompasses the ventral STR and ventromedial prefrontal cortex (PFC), whereas the negative valence network includes the anterior INS and ACC (Fouragnan et al., 2018). Specifically, the INS is a pivotal part of neural architecture underlying the loss aversion (Paulus et al., 2003; Canessa et al., 2013, 2017). We hypothesized that different mechanisms depending on gain and loss domains are involved in risk-taking propensity.

We used computational, neurochemical, and brain-dynamic approaches to examine what factors are associated with decision making for gain and loss domains. In this experiment, participants chose visual meaningless stimuli, i.e., fractal patterns, to maximize their monetary rewards and minimize their losses (Oba et al., 2019, 2021). The trial-and-error process for each participant can be modeled by reinforcement learning (Niv et al., 2012). Thus, reinforcement learning parameters probably reflect individual differences in risk sensitivity to decision making. We constructed three kinds of models to formulate algorithms of trial-and-error processes.

Next, we assessed resting-state levels of γ-aminobutyric acid (GABA) in brain regions using magnetic resonance spectroscopy (MRS). The GABAergic system is critical in several brain functions, such as regulating sensitivity of neurons and orienting focus of attention (Tadin and Blake, 2005). Specifically, MRS studies have demonstrated that GABA levels in the ACC are related to performance of reward-related learning tasks (Scholl et al., 2017; Bezalel et al., 2019). In addition, GABA levels in the ACC and PFC are correlated with performance of Go/No-go tasks (Silveri et al., 2013; Koizumi et al., 2018; Takacs et al., 2021). GABA levels in the left PFC contribute to selective and sustained attention (Kihara et al., 2016; Kondo et al., 2023). The personality trait of lower impulsivity is associated with higher GABA levels in the right PFC (Boy et al., 2011). However, it is still unknown how subcortical GABA levels are linked not only with risk sensitivity, but also with cognitive abilities. On the basis of neuroimaging findings, we postulated that STR and INS GABA levels differ in their involvement in gain and loss domains.

Finally, we investigated dynamics of brain activity as a bridge between behaviors and neurometabolites. A functional-connectivity fMRI study demonstrated that the STR response to monetary reward and loss modulates fronto-parietal activations related to cognitive control (Cubillo et al., 2019). As mentioned above, many brain regions are associated with decision making, but it is still poorly understood how changes in activity patterns contribute to risk-taking propensity. We examined brain states using a novel approach called energy landscape analysis (Ezaki et al., 2017). The advantage of this method lies in a data-driven approach without *a priori* behavioral information, by inferring parameters of the Ising model from given fMRI data. Neuroimaging studies using energy landscape analysis have demonstrated that dynamic transitions of brain states can predict individual differences in perception, attention, and personality traits. For example, transition rates between brain states of the frontal and visual areas are associated with spontaneous switching in visual bistable perception (Watanabe et al., 2014). Furthermore, transition rates between brain states of frontal and parietal areas are correlated with fluctuations of sustained attention (Kondo et al., 2022). Another study showed that people with autism spectrum disorder, relative to healthy people, have infrequent transitions between minor brain states (Watanabe and Rees, 2017). In order to estimate the energy landscape of risk-sensitive networks, we chose regions of interest (ROIs) as follows: the STR, INS, OFC, ACC, amygdala (Amg), and globus pallidus (GP). Brain dynamics were displayed as a series of stays and transitions between different brain states on the energy landscape. We hypothesized that individual differences in risk sensitivity are mediated by brain dynamics based on STR and INS neurotransmission.

## 2 Materials and methods

### 2.1 Participants

Twenty-nine participants were recruited for these experiments. Sample size (*N* = 29) was based on an *a priori* power analysis with a power of 0.8 (*α*-level = 0.05) to detect significant correlations (effect size: *r* = 0.5, bivariate normal model). We computed sample size using G*Power software (ver. 3.1.9.2; Faul et al., 2009). For technical reasons, data from one participant were excluded, leaving 28 participants (13 men and 15 women; mean ± SD age = 26.4 ± 4.8 years, range 20–35 years). They were right-handed with normal or corrected-to-normal vision. None had any history of neurological or psychiatric disorders. This study was approved by the Ethics Research Committee of Chukyo University (approval no. RS18-023) and the Safety Committee of ATR-Promotions (approval no. AN18-052). Experimental procedures were implemented in accordance with Ethical Guidelines for Medical and Biological Research Involving Human Subjects. All participants gave written informed consent after experimental procedures were fully explained to them. Participants were compensated 6,000 yen for their participation.

### 2.2 Behavioral tasks

**Figure 1:**
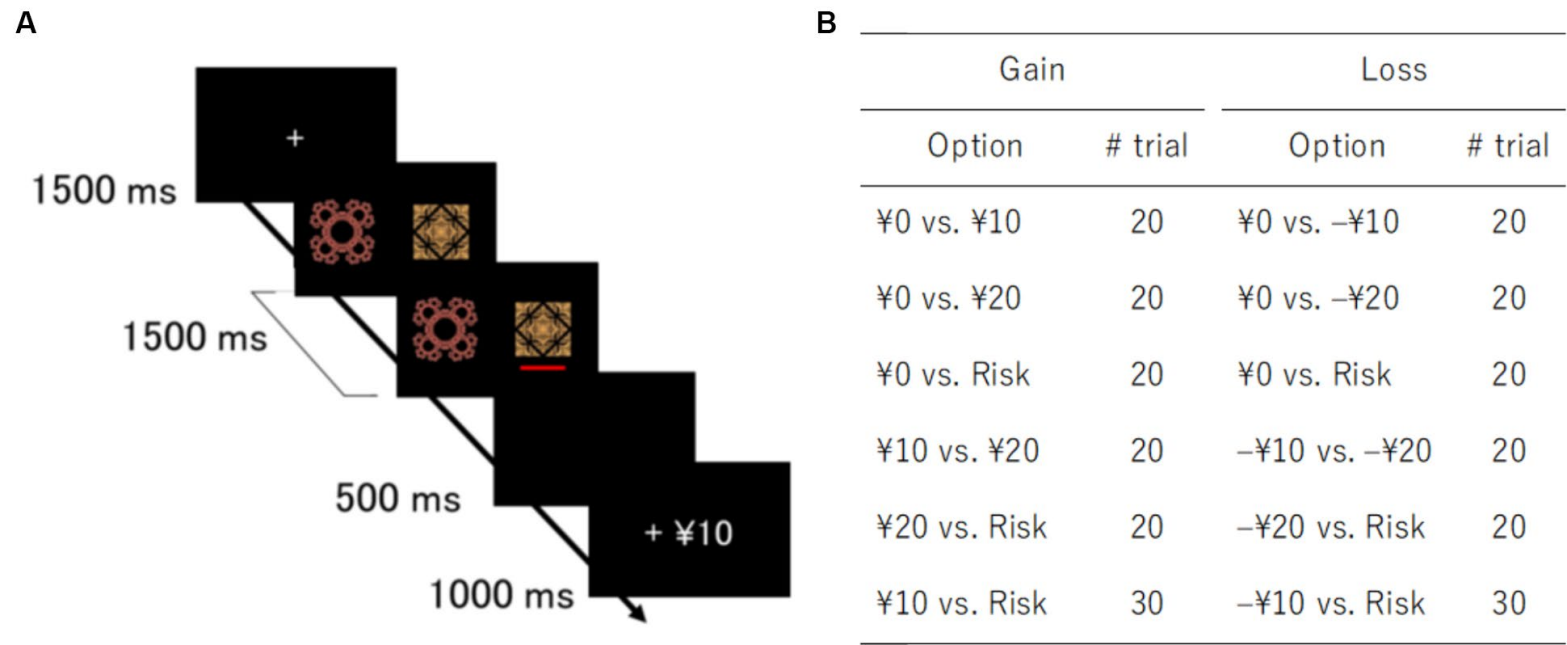
Task design and trial types. **(A)** Schematic representation of a gain trial in a decision-making task. **(B)** Pairs of options and number of trials for gain and loss domains. Risk options yielded random outcomes: 0 or 20 yen in the gain domain and 0 or –20 yen in the loss domain.

Participants performed a behavioral task outside the scanner after imaging data acquisition (Figure 1A). Employing stimuli and task procedures used in previous studies (Oba et al., 2019, 2021), we conducted a psychological experiment. The task consisted of gain and loss domains. The order of the two domains was counterbalanced among participants. Each domain included 10 fractal images as visual stimuli. Different outcomes were randomly assigned to these images.

The gain domain contained three sure options with fixed outcomes (0, 10, and 20 yen) and one variable risky option with random outcomes (0 or 20 yen). Similarly, the loss domain contained three sure options (0, –10, and –20 yen) and one variable risky option (0 or –20 yen). In the risky options, each outcome was randomly chosen with a 50% probability. Each option was paired with the other. All pairs appeared 20 times, except a pair of the risky option and sure option with the same expected value, i.e., 10 yen in the gain domain and –10 yen in the loss domain (Figure 1B). The trial types of 10 yen vs. Risk or –10 yen vs. Risk were presented 30 times to investigate participant risk preference and avoidance. The order of trial types was randomized across participants. They were instructed to maximize monetary rewards and reduce monetary losses. Since participants were not provided with any information about outcome probabilities, they had to learn stimulus-outcome associations throughout the task. Participants received the amount of money earned after the experiment.

For each trial, after a fixation cross disappeared, two fractal images were presented side-by-side on the screen for 1.5 s. Participants were instructed to choose one of the two images with a key press. A red bar was displayed under the chosen image. Feedback regarding outcomes was shown for 1 s after a 0.5-s blank screen. When participants failed to respond within the time window of stimulus presentation, a penalty of –20 yen was imposed. The stimulus position, i.e., right or left, was not related to outcomes. Stimulus presentation and data collection were controlled by a PC with PsychoPy v1.80 (Peirce, 2009). The experiment lasted approximately 30 min.

### 2.3 Behavioral data analyses

We performed a repeated-measure analysis of variance (ANOVA) on learning performance, in which the Greenhouse–Geisser correction was employed to compensate for violations of the sphericity assumption, if needed. For correlation analyses, we computed Pearson’s correlation coefficients with 95% confidence interval (CI). For multiple comparisons of correlation coefficients, we estimated the probability of obtaining the correlation by a random projection of these values. The probability was determined by permuting value vectors 10,000 times and establishing the proportion of random correlations that were higher than the one obtained empirically (*p*_perm_; Kondo et al., 2017). Statistical analyses were carried out with R.^1^

#### 2.3.1 Reinforcement learning models

We used reinforcement learning models to understand the learning process of estimating values of risky options. All models were designed to assign an action value to each action for making decisions. Here, we consider a stimulus *i* on trial *t* for the action value $Q_{t}\left(i\right)$ . The action value for a chosen action was updated based on the following equation (Equation 1):

(1) $$Q_{t+1}\left(i\right)=Q_{t}\left(i\right)+\varepsilon \;\delta_{t}$$

(2) $$\delta_{t}=r_{t}-Q_{t}\left(i\right)$$

where ε is the learning rate that determines the speed of updating the value. The outcome value *r_t_* codes 1 for a gain of 10 yen, 2 for a gain of 20 yen, −1 for a loss of 10 yen, −2 for a loss of 20 yen, or 0 for no gain or loss on trial *t*. δ*_t_* represents the prediction error (PE; Equation 2). Learning proceeds with a decision on each action according to these values, and probabilities of choosing an action are calculated by the softmax function (Equation 3):

(3) $$p_{t}\left(i\right)=\frac{\exp\left(\beta \;Q_{t}\left(i\right)\right)}{\sum_{i'}\exp\left(\beta \;Q_{t}\left(i^{\prime }\right)\right)}$$

where β is a free parameter, inverse temperature, that represents randomness of choice. We refer to this reinforcement learning model as the *standard model*.

In addition to the standard model, we used two models with additional contributions from (i) a different learning rate between positive and negative PEs and (ii) nonlinearity of subjective values for the outcomes. The former is a *separate learning rate model* that accommodates different learning rates for positive PE and negative PE (Equation 4):

(4) $$Q_{t+1}\left(i\right)=\{\begin{array}{c} Q_{t}\left(i\right)+\varepsilon_{P}\;\delta_{t}\;\mathrm{if}\;\delta_{t}>0\\ Q_{t}\left(i\right)+\varepsilon_{N}\;\delta_{t}\;\mathrm{if}\;\delta_{t}<0 \end{array}$$

In this model, if an individual has a larger learning rate for positive PE than for negative PE, one tends to choose risky options (Niv et al., 2012).

The latter is a *subjective utility model* that contains an additional parameter κ, controlling nonlinearly for large outcomes (Equation 5):

(5) $$r_{t}=\{\begin{array}{c} 0\\ 1\\ -1\\ 2\kappa \\ -2\kappa \end{array}\;\begin{array}{l} \mathrm{if}\;\mathrm{the}\;\mathrm{outcome}\;\mathrm{value}\;\mathrm{was}\;0\;\mathrm{yen}\;\mathrm{at}\;\mathrm{trial}\;t\\ \mathrm{if}\;\mathrm{the}\;\mathrm{outcome}\;\mathrm{value}\;\mathrm{was}\;10\;\mathrm{yen}\;\mathrm{at}\;\mathrm{trial}\;t\\ \mathrm{if}\;\mathrm{the}\;\mathrm{outcome}\;\mathrm{value}\;\mathrm{was}\;-10\;\mathrm{yen}\;\mathrm{at}\;\mathrm{trial}\;t\\ \mathrm{if}\;\mathrm{the}\;\mathrm{outcome}\;\mathrm{value}\;\mathrm{was}\;-20\;\mathrm{yen}\;\mathrm{at}\;\mathrm{trial}\;t\\ \mathrm{if}\;\mathrm{the}\;\mathrm{outcome}\;\mathrm{value}\;\mathrm{was}\;-20\;\mathrm{yen}\;\mathrm{at}\;\mathrm{trial}\;t \end{array}$$

When the subjective utility parameter is less than 1, risk tends to be avoided in the gain domain, but accepted in the loss domain (Niv et al., 2012).

#### 2.3.2 Model fitting and comparison

We used a hierarchical type-II maximum likelihood estimation to fit RL models to the data. The fitting procedure was the same as that used in a previous study (Huys et al., 2011). In this method, the marginal likelihood is maximized by the expectation–maximization algorithm in order to estimate hyper parameters of population-level normal distributions. Parameters of learning rate and inverse temperature for each individual were transformed to sigmoid and exponential scale, respectively. We used the Rsolnp package in R^2^ to optimize likelihood functions at the Expectation-step. In the Maximization-step, posterior distributions were estimated by the Laplace approximation to update hyper parameters.

We evaluated the trade-off between parsimony and goodness of fit using integrated Bayesian information criterion (iBIC; Huys et al., 2011). The iBIC assesses the complexity of an evaluated model in terms of the degree of freedom and penalizes more complex models. Smaller iBIC estimates indicate a better fit to the data. We randomly picked up parameter values from population-level distributions and averaged likelihoods of samples for each participant. Averaged likelihoods were transformed to log scale and then summed for all participants.

### 2.4 Imaging data analyses

MRS and fMRI data were obtained at a fixed time between 1:00 and 3:00 p.m. to minimize confounding factors affecting neurometabolite levels. Participants were scanned on a 3-T MRI scanner (MAGNETOM Prisma, Siemens) using a body coil as a transmitter and a 20-channel head coil as a receiver. Small comfortable, elastic pads were placed on both sides of a participant’s head to minimize head motion. Three-dimensional anatomical images of the whole brain were first acquired with a T1-weighted magnetization-prepared rapid gradient echo (MPRAGE) sequence: repetition time (TR) = 2,250 ms; echo time (TE) = 3.06 ms; inversion time = 900 ms; flip angle = 9°; 208 sagittal slices; matrix size = 256 × 256 mm; isotropic voxel size of 1 mm^3^.

**Figure 2:**
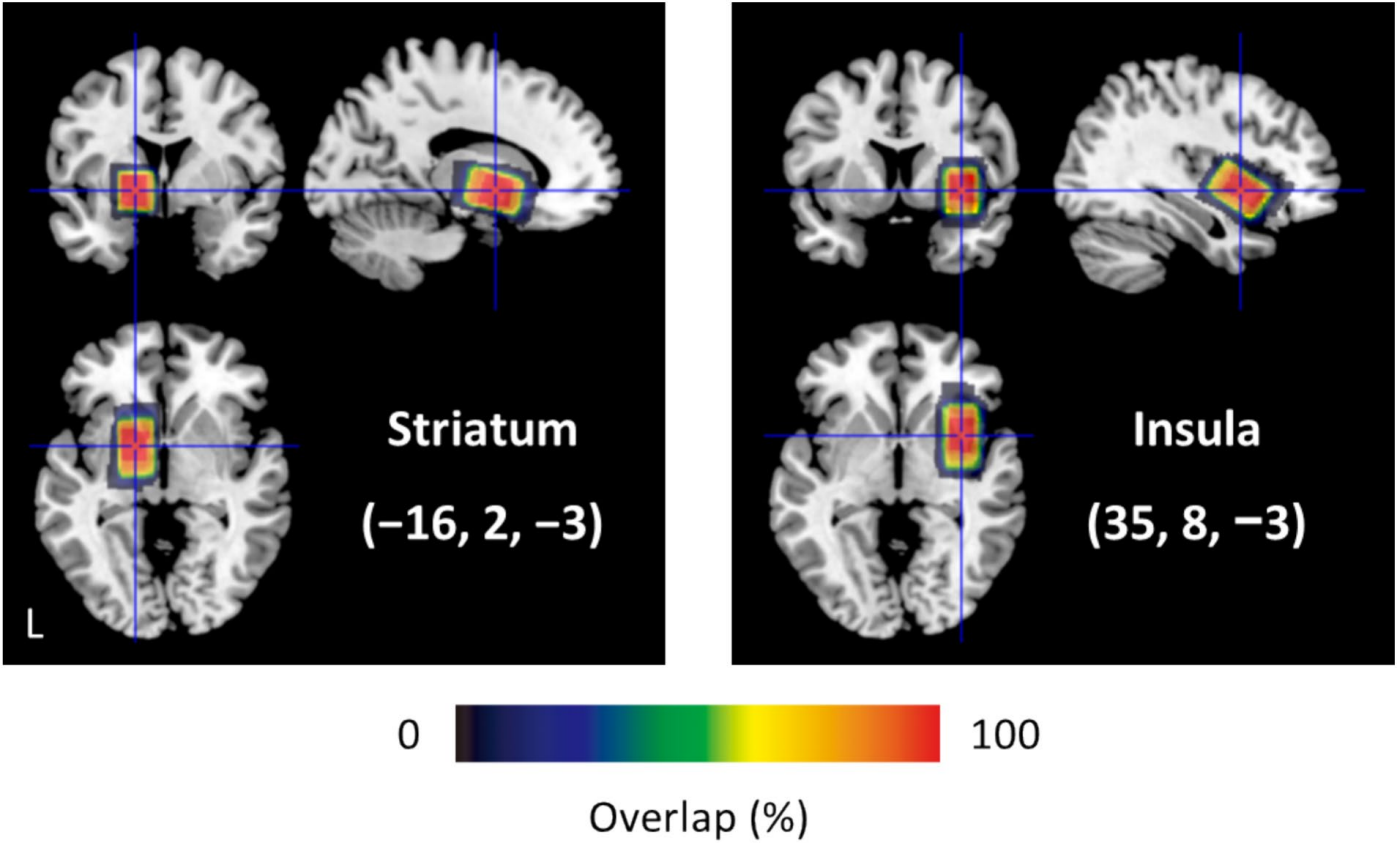
Size and location of MRS voxels (*N* = 28). Centroids of the 20 × 20 × 30 mm^3^ voxels are positioned in MNI coordinates (−16, 2, −3) for the left STR and (35, 8, −3) for the right INS. Each voxel contains gray matter, white matter, and cerebrospinal fluid: 43.2%, 55.0%, and 1.8% for the STR; 68.4%, 16.7%, and 14.9% for the INS.

MRS sessions were conducted before fMRI sessions to avoid gradient-induced frequency drifts (Harris et al., 2014). An MRS session consisted of two runs for the 20 × 20 × 30 mm^3^ voxels, positioned in the left STR and right INS (Figure 2). Locations of the two voxels did not overlap. The STR voxel included the accumbens nucleus, whereas the INS voxel covered the anterior and posterior insular cortices. Voxels were individually tilted to maximize inclusion of gray matter (GM) and minimize white matter (WM) and cerebrospinal fluid (CSF). Using a FASTEST map sequence (Gruetter, 1993; Gruetter and Tkáč, 2000), we performed manual shimming (5 to 10 min) of the magnetic field in the voxel to avoid line broadening. The MEGA-PRESS technique (Mescher et al., 1998) was used to obtain GABA-edited spectra from single-voxel acquisitions: TR/TE = 2000/68 ms; 384/64 measurements, i.e., 192/32 on–off pairs, with/without water suppression; spectral bandwidth of 2 kHz with a sampling rate of 2048 points; editing pulses applied at 1.9 ppm (edit-on) and 7.5 ppm (edit-off). We assessed GABA+ due to co-edited macromolecule contamination. An MRS session lasted approximately 50 min.

In the resting-state fMRI paradigm, 600 volumes were acquired from each participant with closed eyes. The functional images consisted of 45 consecutive slices parallel to the plane of the anterior–posterior commissure and covered the whole brain. A T2*-weighted multiband gradient-echo echo-planar imaging (EPI) sequence was used with the following parameters: TR/TE = 1000/30 ms; flip angle = 50°; multiband acceleration factor = 3; matrix size = 64 × 64; voxel size = 3 × 3 × 3 mm^3^. The fMRI session lasted 10 min.

### 2.5 MRS data analysis

We analyzed MRS data using Gannet 3.0 software (Edden et al., 2014). Preprocessing steps were performed as follows: zero-filling, 3-Hz exponential line broadening, and frequency and phase correction using the spectral registration. We subtracted edit-off spectra from edit-on spectra and used a Gaussian model to estimate neurometabolite measures of a single GABA+ peak at 3.00 ppm. The MRS voxels were segmented into GM, WM, and CSF fractions. For voxel tissue fractions, we corrected institutional units (i.u.) for GABA+/H_2_O by calculating relaxation of water signals in GM, WM, and CSF (Harris et al., 2015). After spatial normalization to Montreal Neurological Institute (MNI) standard space, we computed the overlap of MRS voxels across participants using SPM12^3^ and in-house codes, implemented in MATLAB R2020b (MathWorks, Natick, MA, United States). The criterion of fitting errors (Cramér–Rao lower bounds) was set at less than 20%. Based on an outlier analysis, MRS data of three participants were excluded from subsequent analyses (*N* = 25).

### 2.6 fMRI data analysis

#### 2.6.1 Data preprocessing

We analyzed resting-state fMRI data using SPM12 and DPABI V3.1/DPARSF V4.4^4^ (Verbruggen and Logan, 2008). The 10 initial volumes were discarded from the analysis to achieve steady-state equilibrium between radio-frequency pulsing and relaxation. The remaining 590 volumes were preprocessed. Functional images were calibrated to correct slice acquisition timing and realigned to correct head movement. Movement on the *x*, *y*, and *z* axes was less than 1 mm within each run. Nuisance covariate regression in native space was conducted to minimize head movements using the Friston 24-parameter model (Friston et al., 1996). WM, CSF, and global signals were regressed out to reduce effects of noise caused by cardiac and respiratory cycles and scanner drifts. Functional images were normalized by diffeomorphic anatomical registration using exponentiated Lie algebra (DARTEL), resampled to a voxel size of 3 × 3 × 3 mm^3^, and smoothed with an isotopic Gaussian kernel of 4-mm full-width at half-maximum. Data were band-pass filtered at 0.01 to 0.1 Hz. We extracted blood oxygen level dependent (BOLD) signals from the automated anatomical labeling atlas (Tzourio-Mazoyer et al., 2002). On the basis of our hypotheses, we focused on data of the following eight ROIs: the OFC, medial OFC, INS, ACC, Amg, caudate (Cd), putamen (Pu), and GP. The Cd and Pu are included in the STR. Averaged data of both hemispheres for each participant were used in the subsequent analyses.

#### 2.6.2 Energy landscape analysis

We performed the energy landscape analysis in essentially the same way as a previous study (Ezaki et al., 2018). We binarized BOLD signals for each ROI. Appearance probabilities of brain activity patterns were fitted with the pairwise maximum entropy model, i.e., Boltzmann distribution. Using “energy” values defined in the fitting function, we constructed the energy landscape representation of activity patterns. On the basis of the energy landscape, we divided activity patterns into discrete states, each of which corresponds to a basin of a local minimum. Finally, using the list of activity patterns in each discrete state, we obtained a coarse-grained representation of the original time series. We describe these procedures in detail in the following subsections.

#### 2.6.3 Pairwise maximum entropy model

For each participant and each ROI, we computed the average value of the BOLD signal, which was then used as a threshold to binarize the signal into −1 or + 1, i.e., inactive or active. For each volume, the brain state was represented by an activity pattern $\sigma =\left(\sigma_{1},\sigma_{2},\;\ldots ,\;\sigma_{8}\right)$ where $\sigma_{i}$ $\left(i=1,\;\ldots ,\;8\right)$ denotes the activity of *i*th ROI (inactive: $\sigma_{i}=-1$ , active: $\sigma_{i}=1$ ). We computed the appearance probability of each of $2^{8}\left(=256\right)$ activity pattern in these data. This empirical probability distribution was fitted with the pairwise maximum entropy model (Equation 6):

(6) $$P\left(\sigma \right)=\frac{\exp\left(-E\left(\sigma \right)\right)}{\sum_{\sigma }\exp\left(-E\left(\sigma \right)\right)}$$

where $E\left(\sigma \right)=-\sum_{i=1}^{8}h_{i}\sigma_{i}-\sum_{i=1}^{8}\sum_{j=1}^{8}J_{ij}\sigma_{i}\sigma_{j}$ denotes an energy value defined for each activity pattern. We tuned parameters of the model, i.e., $h_{i}$ and $J_{ij}$ using the gradient ascent algorithm to maximize the likelihood function (Ezaki et al., 2017). The accuracy of fitting (r_D_) was sufficiently high (r_D_ = 0.98). We use the energy value defined for each activity pattern, $E\left(\sigma \right)$ , in the following analyses.

#### 2.6.4 Construction of the energy landscape

Here we define two activity patterns $\left(\sigma =\right)\alpha$ and $\left(\sigma =\right)\beta$ as neighbors, if these two patterns differ at a single ROI, that is, if the Hamming distance between these activity patterns is equal to 1. Thus, each of the 256 activity patterns has eight neighbors. The energy landscape was constructed as follows. (i) We selected an activity pattern. (ii) We moved to one of its neighboring activity patterns, which had a minimum energy value in the neighbors and the original activity pattern. (iii) We repeated (ii) until it was trapped in a local minimum. (iv) We recorded this path. (v) Procedures (i) to (iv) were repeated over all initial activity patterns. The resultant paths defined sets of activity patterns belonging to basins of local minima. This way, for each activity pattern, a single local minimum to which it belongs was identified. Note that if equally steep paths were found in (ii), activity patterns could fall into more than one local minimum, but this did not occur in our analysis.

## 3 Results

### 3.1 Behavioral results

**Figure 3:**
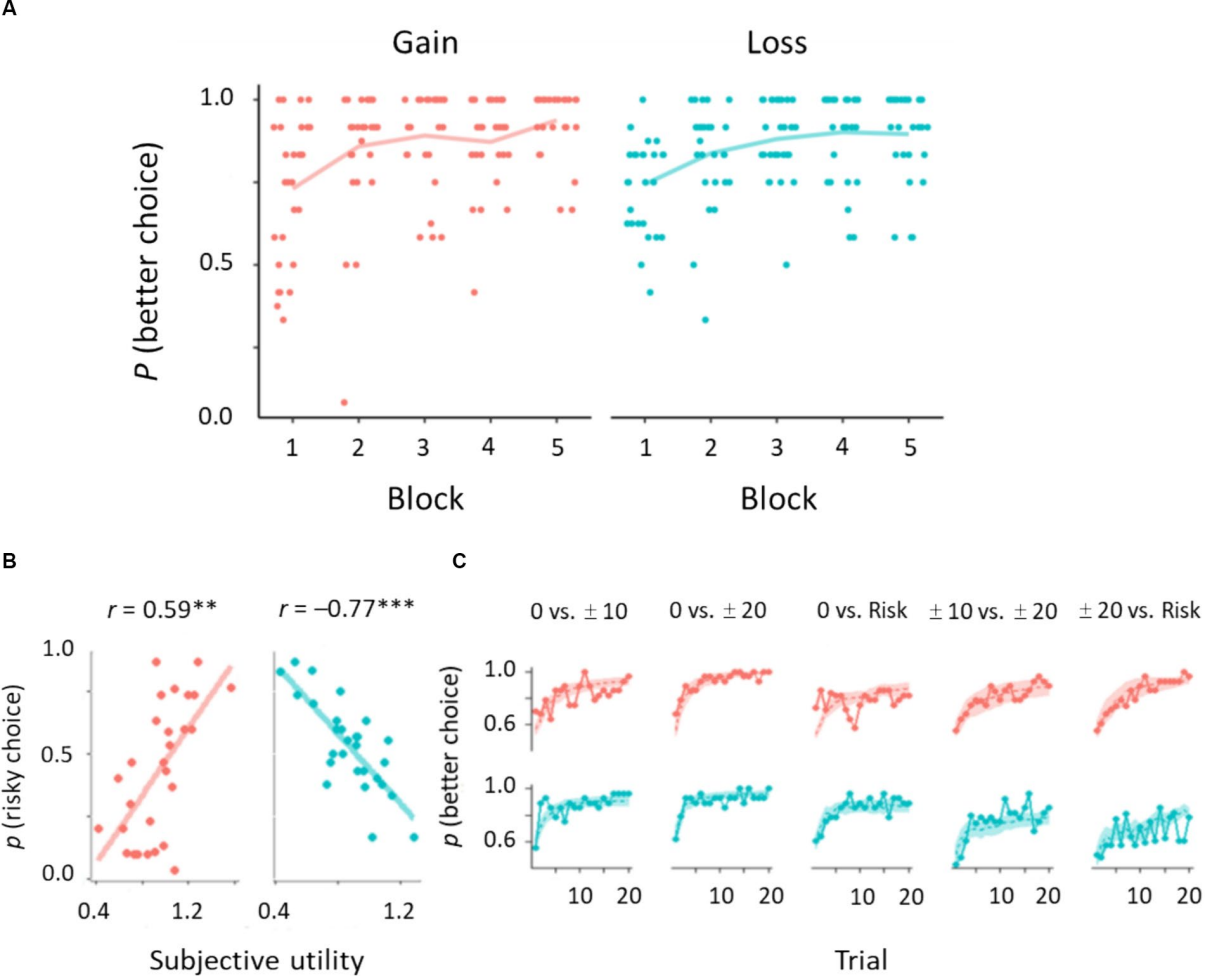
Behavioral results of gain and loss domains. **(A)** Learning curves of choosing better options. Dots indicate individual data (*N* = 28). **(B)** Correlations between subjective utility parameters and the proportion of risky choices. **(C)** Learning performance derived from experimental and simulated data. Solid lines indicate the proportion of better choices averaged across participants. Shaded areas represent 95% confidence intervals of the prediction based on the subjective utility model. ***p < 0.001, **p < 0.01.

We first checked participant learning performance in trials of surely better options: 0 vs. 10, 0 vs. 20 yen, and 10 vs. 20 yen for the gain domain; 0 vs. −10 yen, 0 vs. −20 yen, and −10 vs. −20 yen for the loss domain (Figure 1B). Learning curves for each domain are shown in Figure 3A. A trend test demonstrated that learning performance increased gradually over time: Jonckheere–Terpstra test, *T*_JT_ = 9.43, *p* < 0.01 for the gain domain; *T*_JT_ = 9.74, *p* < 0.01 for the loss domain. We performed a 2 (domain) × 3 (trial type) ANOVA on learning better choices. These results showed that the proportion of better choices (mean ± SD) did not differ between the gain (0.856 ± 0.182) and loss (0.850 ± 0.158) domains: *F*(1, 27) = 0.06, *p =* 0.80, $\eta_{p}^{2}$ = 0.002. The proportion of better choices was greater for the trials of 0 vs. 20 yen and 0 vs. −20 yen (0.923 ± 0.101) than for the other two trial types; 0 vs. 10 yen and 0 vs. −10 yen (0.853 ± 0.167); 10 vs. 20 yen and − 10 vs. −20 yen (0.782 ± 0.199); *F*(2, 54) = 11.57, *p* < 0.001, $\eta_{p}^{2}$ = 0.30. After Bonferroni correction, for all trial types, the proportion of better choices was greater than chance level (50%): *t* > 6.87, *p* < 0.001, Cohen’s *d* > 1.30. The interaction between domain and trial type was not significant: *F*(2, 54) = 2.08, *p* = 0.16, $\eta_{p}^{2}$ = 0.07. Thus, participants succeeded in learning better choices, regardless of domains.

Next, we investigated the learning performance on risky options: 0 yen vs. Risk, 20 yen vs. Risk, and 10 yen vs. Risk for the gain domain; 0 yen vs. Risk, −20 yen vs. Risk, and −10 yen vs. Risk for the loss domain. We conducted a 2 × 3 ANOVA on choosing risky options. Overall risk preference did not differ between the gain domain (0.461 ± 0.326) and the loss domain (0.449 ± 0.269): *F*(1, 27) = 0.13, *p* = 0.71, $\eta_{p}^{2}$ = 0.01. The main effect of trial types was significant: 0 yen vs. Risk (0.466 ± 0.349) and ± 10 yen vs. Risk (0.484 ± 0.232) > ±20 yen vs. Risk (0.414 ± 0.303), *F*(2, 54) = 4.09, *p* = 0.03, $\eta_{p}^{2}$ = 0.13. The interaction was also significant: *F*(2, 54) = 127.16, *p* < 0.001, $\eta_{p}^{2}$ = 0.82. In the gain domain, we found the following pattern of risk preference: 0 yen vs. Risk (0.781 ± 0.128) > 10 yen vs. Risk (0.434 ± 0.270) > 20 yen vs. Risk (0.167 ± 0.204). We found the reverse pattern in the loss domain: −20 yen vs. Risk (0.662 ± 0.136) > −10 yen vs. Risk (0.534 ± 0.179%) > 0 yen vs. Risk (0.152 ± 0.162). These results indicate that participants could accurately assess risk options in this task because expected values of the risk option were 10 yen for the gain domain and −10 yen for the loss domain.

### 3.2 Model selection

We conducted a model selection analysis at the population level. In the gain domain, iBIC estimates were smaller for the subjective utility model (2952.72) than for the standard model (3002.78) and separate learning rate model (2960.07). In the loss domain, iBIC estimates were smaller for the subjective utility model (3435.17) than for the standard model (3491.75) and separate learning rate model (3499.87). These results indicate that a participant decision is well explained by nonlinear evaluation of outcomes to guide his/her own choices during the risky trials. The subjective utility parameter was correlated with the proportion of risky choices: *r* = 0.59, *p* = 0.004, 95% CI [0.30, 0.82] for the gain domain; *r* = −0.77, *p* < 0.001, 95% CI [−0.90, −0.59] for the loss domain (Figure 3B). The subjective utility parameter also showed significant correlations with sure options with small and large outcomes: *r* = 0.51, *p* = 0.006, 95% CI [−0.75, −0.14] for the gain domain; *r* = −0.59, *p* < 0.001, 95% CI [−0.80, −0.25] for the loss domain. The learning curve yielded by the subjective utility model was in good agreement with the population average for most cases, including the better options (Figure 3C).

### 3.3 MRS results

We obtained neurometabolite measures from MRS voxels for each participant (Figure 2). GABA+ levels (mean ± SD) were 2.95 ± 0.65 i.u. for the STR; 3.42 ± 0.62 i.u. for the INS. Using Shapiro–Wilk tests, we determined that MRS data followed a normal distribution: *W* > 0.949, *p* > 0.24. Smirnov–Grubbs tests did not indicate any outliers of MRS data. Fitting errors for GABA+ levels were 9.6 ± 3.9% for the STR; 8.1 ± 2.2% for the INS. Thus, the quality of MRS data was satisfactory.

**Figure 4:**
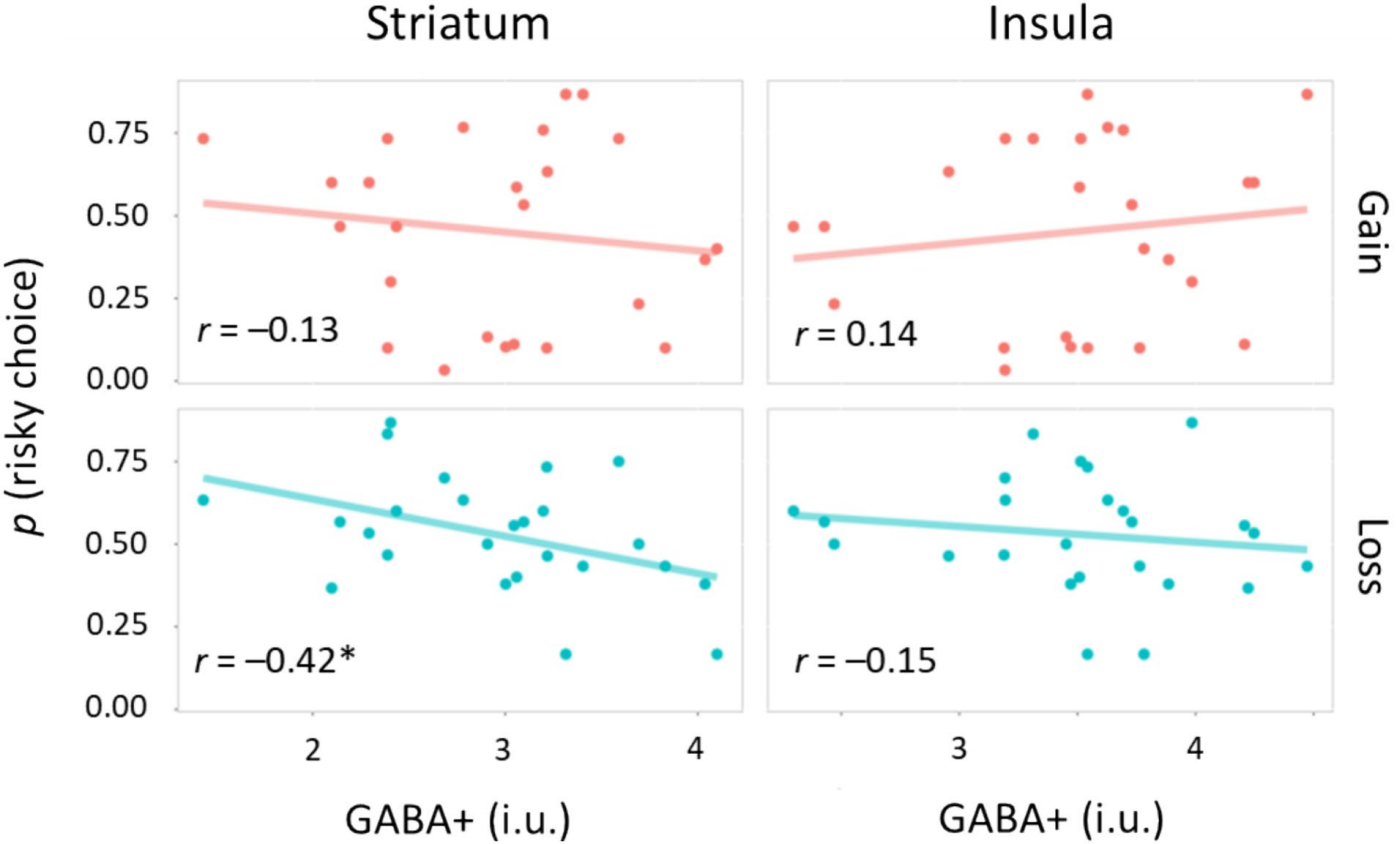
Correlations between neurometabolite and risk sensitivity. Scatter plots for the relationship between GABA+ levels and the proportion of risky choices. Circles indicate individual data, whereas solid lines represent linear regression fits. i.u., institutional unit *p < 0.05.

We investigated whether neurometabolite measures were associated with risk sensitivity during the learning task (Figure 4). For the gain domain, there was no correlation between the proportion of risky choices and GABA+ levels: *r* = −0.13, *p* = 0.52, 95% CI [−0.50, 0.28] for the STR; *r* = 0.14, *p* = 0.51, 95% CI [−0.27, 0.51] for the INS. However, we found an intriguing pattern of correlations for the loss domain. The proportion of risky choices was negatively correlated with GABA+ levels in the STR (*r* = −0.42, *p* = 0.038, 95% CI [−0.70, −0.03]), but not with those in the INS (*r* = −0.15, *p* = 0.47, 95% CI [−0.52, 0.26]). When we performed multiple comparisons of four correlation coefficients, the correlation between risky loss choices and STR GABA+ levels was marginally significant: *p*_perm_ = 0.089. Thus, GABA+ levels in the STR are involved in risk preference/avoidance, particularly in the loss domain.

### 3.4 fMRI results

**Figure 5:**
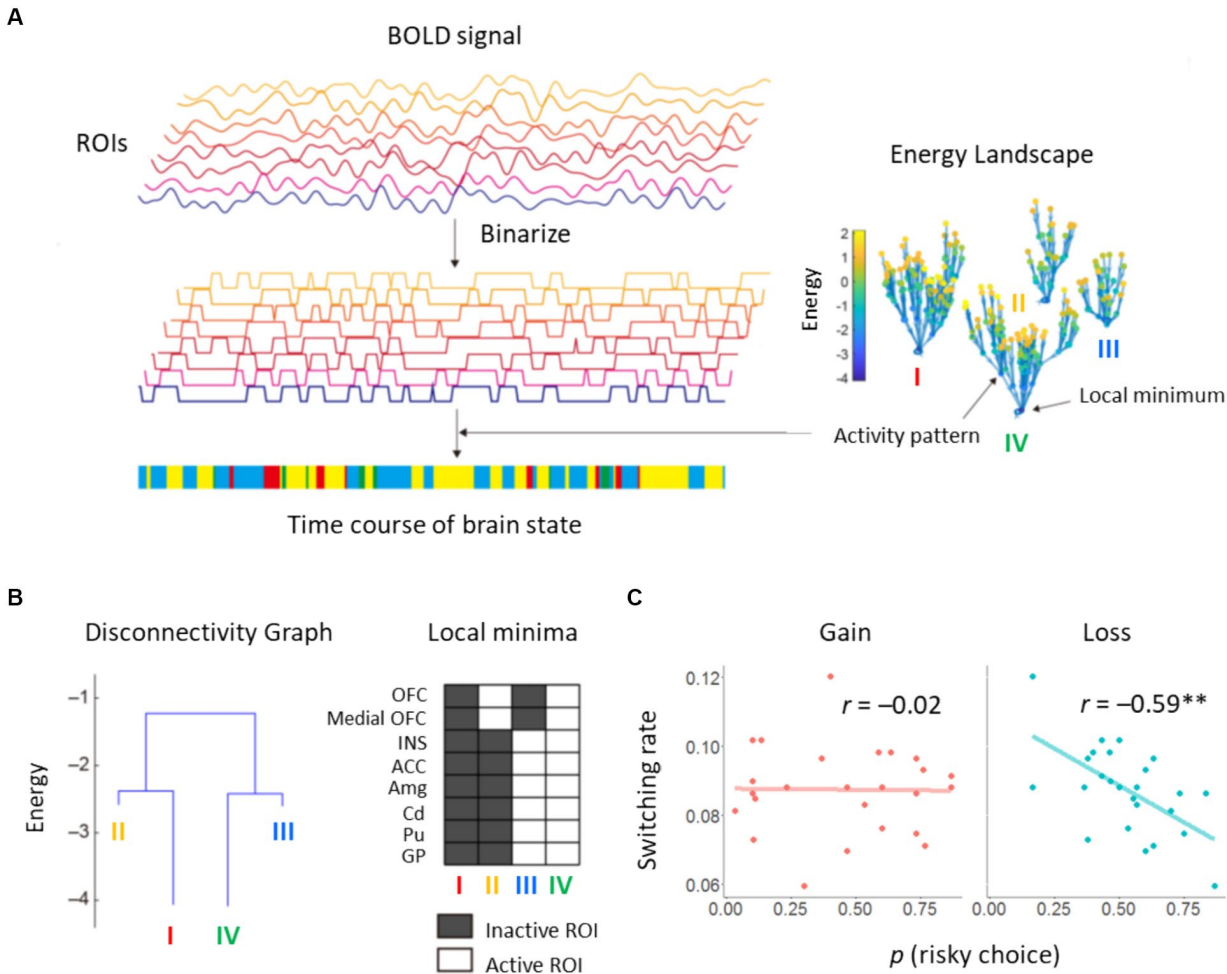
Results of energy landscape analysis. **(A)** BOLD signals obtained from eight ROIs were binarized. Appearance probability of each binarized state, i.e., activity pattern, was fitted by pairwise maximum entropy model. Using the energy value in the fitted function, an energy landscape was constructed. Activity patterns were categorized into basins of local minima. The brain state at each volume was characterized by a basin in the energy landscape. **(B)** Constructed disconnectivity graph. The switching rate between brain states was derived from activity patterns of active and inactive ROIs. **(C)** Correlations between the proportion of risky choices and the switching rate.

Computation of the energy landscape was motivated to bridge behavioral and neurometabolite factors. Construction of an energy landscape resulted in 256 possible activity patterns being classified into four basins, i.e., brain states (Figure 5A). Two synchronized activity patterns appeared most frequently in these data and constituted major brain states in the energy landscape (states I/IV in Figure 5B). The other two minor brain states were also found as asynchronized patterns between the OFC/medial OFC and other ROIs (states II/III in Figure 5B). We calculated switching rate between two major states. It should be noted that this switching rate between brain states is a commonly used measure in the literature of energy landscape analysis (Ezaki et al., 2018). These suggest coordinated dynamics among the ROIs. We focused on brain dynamics involving the two major basins with synchronized activity patterns. We investigated whether risk sensitivity for each participant was explained by the switching rate between active and inactive states. For the loss domain, we found a negative correlation between risk preference and switching rate: *r* = −0.59, *p* = 0.002, 95% CI [−0.80, −0.25] (Figure 5C). Specifically, the subjective utility parameter κ was correlated with switching rate: *r* = 0.67, *p* < 0.001, 95% CI [0.38, 0.84]. Correlations between the other parameters and switching rate did not reach statistical significance: *r* = 0.30, *p* = 0.14, 95% CI [−0.10, 0.62] for the learning rate ε ; *r* = −0.37, *p* = 0.069, 95% CI [−0.67, 0.03] for the inverse temperature β. No correlation was found for the gain domain: *r* = −0.02, *p* = 0.94, 95% CI [−0.41, 0.38] (Figure 5C). These results indicate that dynamic transitions of brain states, including the STR and INS, reflect individual propensity for risk learning process, particularly in the loss domain.

## 4 Discussion

We showed mechanisms of risk sensitivity at the computational, neurochemical, and brain dynamic levels. Participant trial-and-error processes were successfully modeled by reinforcement learning. Specifically, the subjective utility model better accounted for participant learning performance than the standard and separate learning rate models. MRS results demonstrated that participants with higher GABA+ levels in the STR had a risk-avoidance propensity in the loss domain. Energy landscape analyse were performed to characterize multivariate dynamics of risk-sensitive brain activations. These results revealed that frequent switching between dominant brain states was associated with risk avoidance in the loss domain. We discuss these issues in turn.

The subjective utility model better fit the obtained data than the other models. Experience-based decision-making under risk is related to signed PEs (Niv et al., 2012; Lefebvre et al., 2017; Moeller et al., 2021). Specifically, previous studies demonstrated a significant correlation between subjective utility parameters and individual risk sensitivity (Niv et al., 2012; Oba et al., 2021). Our results showed that the subjective utility model predicted not only individual risk sensitivity, but also learning performance of choice between sure options with large and small outcomes. This is because the subjective utility parameter is the weight for the large outcome across the risky and sure options. In contrast, the separate learning rate model represents differences in the effect of PE on value updating, and in the current learning task, differences in the effect of PE occur only in the risk option; thus, the extent to which it can predict learning performance was limited in the separate learning rate model. Therefore, we believe that the subjective utility model was superior in explaining both sure and risky choices.

GABA+ levels in the STR were negatively correlated with the proportion of risky options in the loss domain, but was not in the gain domain. The asymmetry of these results may reflect a general tendency that people usually take risks in the loss domain, but not in the gain domain (Kahneman and Tversky, 1979). Gain-related learning is supported by the reward system, which is based on dopaminergic projections from brainstem nuclei to the STR and frontal areas (Conio et al., 2020). An early study demonstrated that the phasic activity of dopamine neurons encodes the reward PE signal in reinforcement learning (Schultz et al., 1997). A value function of learning is represented as neural plasticity, i.e., synaptic strength, that is regulated by released dopamine (Reynolds et al., 2001; Yagishita et al., 2014). On the other hand, neural mechanisms of loss-related learning are still unclear. Our results suggest that GABA+ levels in the STR can shape individual risk sensitivity to monetary loss.

Neuroimaging studies have demonstrated that the anterior part of the INS is activated during anticipation of pain (Chua et al., 1999; Ploghaus et al., 1999), but also during risky choice in games (Paulus et al., 2003; Canessa et al., 2013; Cui et al., 2022). However, we did not find a significant correlation between GABA+ levels in the INS and risk sensitivity. Thus, the INS may have functional dissociations between neurometabolite and brain activity levels. A few substantial MRS studies have investigated whether GABA+ levels in the dorsal part of the ACC affect probabilistic learning performance. Using a reward-related learning paradigm, higher Glx (glutamate–glutamine) and lower GABA+ levels were independently linked with better learning performance (Scholl et al., 2017). In contrast, another study showed that GABA+ levels were positively correlated with monetary gain and loss in auditory discrimination learning, but Glx levels were not (Bezalel et al., 2019). This discrepancy may be due to differences in cognitive demand and task complexity (Li et al., 2022). However, it should be noted that risk sensitivity observed in this study is not intrinsically related to good or bad performance, but rather to risk preference or avoidance for each individual.

Our energy landscape analysis classified activity patterns of the eight ROIs into two major and two minor brain states. Intriguingly, activity patterns differed between the OFC and the other regions, including the STR, INS, and ACC. Each brain region has been identified as relevant to decision-making and learning under risk, but may have separate functions. For instance, it is assumed that STR activity codes PE signals (Niv et al., 2012) and OFC activity represents action values (O'Doherty et al., 2021). Thus, representations integrated by different activities of ROIs may represent non-linear, subjective loss values. The switching rate between the two major states was negatively correlated with the proportion of risky options in the loss domain (*r* = −0.59). This large effect size indicates that even activity patterns of some ROIs could represent essential parts of whole-brain activity in risk learning. Specifically, the switching rate was closely linked to the subjective utility parameter κ in the loss domain (*r* = 0.67). This suggests that dynamic transitions of brain states are directly involved in nonlinearity of the subjective value of money.

Previous studies using energy landscape analysis have revealed that dynamic transitions of brain states can predict individual differences in perception and attention. Perceptual switching in a structure-from-motion illusion was associated with the switching rate to frontal-area or visual-area major states (Watanabe et al., 2014). In addition, fluctuations of attention levels were correlated with the switching rate between different brain states derived from the dorsal-attention and default-mode networks (Kondo et al., 2022). Taking these findings into account, different ROIs may also have different energy landscapes that are compatible with the gain domain. We provide the first evidence that brain dynamics of ROIs can predict individual variability of risk preference and avoidance, particularly in the loss domain.

This research identified differences in risk-learning brain functions between gain and loss domains: (i) the subjective utility model explains participant propensity for risk sensitivity; (ii) GABA+ levels in the STR are correlated with risk preference/avoidance in the loss domain; and (iii) frequent switching between subcortical brain states is associated with risk avoidance in the loss domain. Our findings suggest a close linkage between decision making under risk, risk-sensitive brain dynamics, and striatal GABA levels. However, we were unable to determine which neurometabolite levels are involved in the gain domain. Previous studies indicate that the dopaminergic system contributes to risk sensitivity in the gain domain. Thus, future studies should further investigate neural mechanisms related to gain and loss domains by coupling behavioral analyses with multimodal neuroimaging, namely, fMRI and MRS. However, it should be noted that the present results show a stable propensity in the loss domain for each individual. Exploration of mechanisms underlying risk-taking tendencies and decision-making processes is crucial not only for the treatment of addiction and anxiety disorders, but also for marketing strategies (Opris et al., 2020).

## Data availability statement

The original contributions presented in the study are included in the article/Supplementary material, further inquiries can be directed to the corresponding author.

## Ethics statement

The studies involving humans were approved by the Research Ethics Committee of Chukyo University. The studies were conducted in accordance with the local legislation and institutional requirements. The participants provided their written informed consent to participate in this study.

## Author contributions

HMK: Conceptualization, Data curation, Formal analysis, Funding acquisition, Investigation, Project administration, Resources, Visualization, Writing – original draft, Writing – review & editing. TO: Conceptualization, Data curation, Formal analysis, Resources, Software, Visualization, Writing – original draft, Writing – review & editing. TE: Conceptualization, Data curation, Formal analysis, Funding acquisition, Resources, Software, Visualization, Writing – original draft, Writing – review & editing. TK: Data curation, Formal analysis, Resources, Software, Visualization, Writing – original draft, Writing – review & editing. YS: Data curation, Investigation, Resources, Writing – review & editing. HO: Conceptualization, Funding acquisition, Resources, Supervision, Writing – review & editing.

## Footnotes

^1^https://www.r-project.org/

^2^https://cran.r-project.org/package=Rsolnp

^3^http://www.fil.ion.ucl.ac.uk/spm

^4^http://rfmri.org/dpabi
